# A Model-Agnostic Meta-Baseline Method for Few-Shot Fault Diagnosis of Wind Turbines

**DOI:** 10.3390/s22093288

**Published:** 2022-04-25

**Authors:** Xiaobo Liu, Wei Teng, Yibing Liu

**Affiliations:** 1 Key Laboratory of Power Station Energy Transfer Conversion and System, North China Electric Power University, Ministry of Education, Beijing 102206, China; liuxiaobo116@163.com (X.L.); tengw@ncepu.edu.cn (W.T.); 2 Hebei Key Laboratory of Electric Machinery Health Maintenance & Failure Prevention, North China Electric Power University, Baoding 071003, China

**Keywords:** few-shot learning, fault diagnosis, model-agnostic meta-learning, wind turbines

## Abstract

The technology of fault diagnosis is helpful to improve the reliability of wind turbines, and further reduce the operation and maintenance cost at wind farms. However, in reality, wind turbines are not allowed to operate with faults, so few fault samples could be obtained. With a small amount of training data, traditional fault diagnosis models that need huge samples under a deep learning framework are difficult to maintain with high accuracy and effectiveness. Few-shot learning can effectively solve the problem of overfitting caused by fewer fault samples in model training. In view of model-agnostic meta-learning (MAML), this paper proposes a model for few-shot fault diagnosis of the wind turbines drivetrain, which is named model-agnostic meta-baseline (MAMB). The training data is input to the base classification model for pre-training, then, some data is randomly selected from the training set to form multiple meta-learning tasks that are utilized to train the MAML to finally fine-tune the later layers of the model at a smaller learning rate. The proposed model was analyzed by the small samples of the bearing data from Case Western Reserve University (CWRU) data, the generator bearings, and gearboxes vibration data in wind turbines under randomly changing operating conditions. The results verified that the proposed method was superior in one-shot, five-shot, and ten-shot tasks of wind turbines.

## 1. Introduction

As the installed capacity of wind turbines increases rapidly, the technology of condition monitoring and fault diagnosis attracts more attention to guarantee the operational reliability of wind turbines. The huge vibration data collected from wind farms prompts the development of intelligent diagnosis of wind turbines, which is driven by the progress of the technology of artificial intelligence. However, in reality, wind turbines are not allowed to run with faults. When a fault occurs, the wind turbine has to shut down. Therefore, the collected operating data are mostly normal data under healthy status, with very few fault data. Obviously, these kinds of data from wind turbines are insufficient to train an intelligent classification model using traditional deep learning, due to the potential overfitting caused by sample imbalance and type imbalance. Data augmentation and regularization techniques can alleviate the overfitting caused by low data volume [1]. Data augmentation refers to the addition of data by manual rules such as pan, flip, cut, and rotate. Designing these rules relies heavily on domain knowledge and requires expensive labor costs. Regularization can be used to correct the direction of descent. However, neither of the two methods can fundamentally solve the overfitting problem when data are extremely scarce.

Few-shot learning can train deep models with very limited data and solve the problem of overfitting caused by a small number of fault samples. The current few-shot learning makes full use of the advantages of deep neural networks in feature representation and end-to-end model optimization to solve the overfitting problem from different perspectives, e.g., generative modeling, metric learning, and meta-learning [2].

Generative modeling is an intuitive method to increase the number of training samples and enhance the diversity of data. Generative adversarial networks (GAN) have been widely used in recent years due to their excellent performance. Hu et al. proposed a data augmentation algorithm based on order tracking and a self-adaptive convolutional neural network for fault diagnosis [3]. Zheng et al. achieved data augmentation by improving GAN to enhance the accuracy of imbalance fault diagnosis [4]. However, when data is very scarce, generative modeling cannot be trained well and may suffer from pattern collapse, which in turn leads to poor results.

The main goal of metric learning is to learn a similarity metric under the circumstance where a pair of similar samples can obtain a higher similarity score while nonsimilar pairs obtain a lower similarity score. Ren et al. proposed a capsule autoencoder model based on a capsule network for intelligent fault diagnosis of few shots [5]. Based on metric learning, siamese neural networks [6,7], neural networks with external memories [8,9], relation networks [10], and graph neural networks [11] have been successfully applied to few-shot learning. The results of metric learning depend on the sampling strategy. If the sampling strategy is too simple, only simple samples will be learned; if the sampling difficulty is too high, it will lead to slow convergence, nonconvergence, or even overfitting.

Meta-learning, also known as “learning to learn”, is similar to the way that humans learn by analogy and inference. Meta-learning attempts to improve the network’s ability to learn higher-level tasks, rather than only classification tasks, by learning the feature representation of the task and generalizing on new tasks. Few-shot learning is a concrete application of meta-learning in the field of supervised learning. Hospedales et al. provide a detailed overview of meta-learning from various aspects including research areas, algorithm improvements, and application challenges [12]. Currently, meta-learning has been widely used in the field of image recognition [13,14,15,16]. Specifically in meta-learning, it randomly selects a series of few-shot classification tasks from training samples, extracts general knowledge as additional information, and optimizes the model to perform well on testing tasks, which can effectively solve the problem of overfitting in training a learning model with few samples.

Meta-learning-based few-shot learning has been gradually applied in the field of fault diagnosis. Wu et al. constructed seven few-shot transfer learning methods based on 1D convolutional networks based on meta-learning [17]. Wang et al. proposed a meta-learning model in the light of a feature space metric for fault diagnosis of bearings [18]. Feng et al. proposed a semi-supervised meta-learning network with squeezed incentive attention for low-probability fault diagnosis [19]. Su et al. presented a novel method called data reconstruction hierarchical recurrent meta-learning for bearing fault diagnosis under different working conditions [20]. Wang et al. proposed a metric-based meta-learning method named Reinforce Relation Network for bearing fault diagnosis [21].

The aforementioned meta-learning-based fault diagnosis mainly aimed at the testbed data under certain working conditions and realized the transfer from one fixed working condition to another working condition. However, the actual wind turbine operating conditions vary randomly, and compound failures often occur. The fault diagnosis of wind turbines is of great importance. As an innate monitoring system equipped in wind turbines, supervisory control and data acquisition (SCADA) cover a wide range of subassemblies by abundant monitoring parameters, e.g., wind speed, rotational speed, vibration, current, voltage, wind power, etc. Encalada et al. proposed a predictive model using only SCADA data, which can work under different and varying operating and environmental conditions [22]. Castellani et al. detected anomalies in damaged wind turbines based on the novelty index of the Mahalanobis distance [23]. Meyer et al. proposed a new fault diagnosis method that combines autonomous data-driven learning of fault signatures and health state classification based on convolutional neural networks and isolation forests [24]. Artigao et al. identified the frequency components associated with a fault from the current spectrum of a faulty wind turbine motor and compares it with the current spectrum of a healthy motor to achieve fault diagnosis [25].

SCADA-based time series for anomaly detection in wind turbines is of great practical importance [26,27,28,29,30,31,32,33,34]. However, there are currently some limitations in using SCADA for fault diagnosis of wind turbines. SCADA cannot monitor vibrations at multiple measurement points and locations as a wind turbine condition monitoring system (CMS) can. Very precise fault location of wind turbines is not possible with SCADA data, for example, SCADA data cannot determine whether the inner or outer ring of a bearing is faulty or detect compound faults in multiple gears of a gearbox. The use of CMS can solve the limitations of SCADA and can effectively diagnose the specific fault location.

Our group has done a range of work on fault diagnosis for wind turbines using CMS. In the literature [35] the complex wavelet transform was used to extract weak faults in the wind turbine gearbox by analyzing the strips of the multiscale enveloping spectrogram (MuSEnS) on different scales. Conventional demodulation analysis, cyclic coherence function, complex wavelet transform, and spectral kurtosis were used to analyze the vibration signals of a real 2 MW wind turbine generator with a faulty bearing [36]. Empirical wavelet transform was utilized to adaptively find weak fault frequency in the planetary stage as well as evident fault characteristics in other ordinary stages [37]. The normalized multi-stage enveloping spectrogram was presented to reveal the fault characteristic frequencies of planetary gears and bearings [38]. The literature [39] reviewed almost all the research on the vibration-based diagnosis algorithm for wind turbines in the past decade.

The above research mainly addresses the problem of variable operating conditions, compound faults and weak faults in wind turbines from the perspective of signal processing. Signal processing often requires some prior knowledge and expert experience. In contrast, deep learning does not require too much human intervention and can effectively improve the intelligence of fault diagnosis. CMS is more expensive and requires additional hardware and software costs. Therefore, in reality, CMS does not have access to sufficient sample data as SCADA does. In practical situations, wind turbine fault samples are few, and the specific operating conditions at every moment cannot be accurately obtained. Therefore, wind turbines’ few-shot learning requires a more powerful meta-learning model. In combination with convolutional neural network (CNN) pre-training, MAML, and fine-tuning, this paper makes full use of CNN’s classification ability, MAML’s generalization ability to learn new tasks, and fine-tuning’s ability to further optimize parameters, so as to better solve the problem of wind turbine few-shot fault diagnosis under variable working conditions and noise.

In this paper, a novel few-shot fault diagnosis model of wind turbine drivetrain based on model-agnostic meta-learning (MAML) is carried out. Three types of vibration data are analyzed to verify the advantages of the proposed model, including the few-shot case of the bearing data from Case Western Reserve University (CWRU), the few-shot case of wind turbine generator bearing, and the few-shot case of wind turbine gearbox. Each class of data contained data in both x and y directions, all sampled at 1 s. All training data was input into the classifier to train a base model, i.e., the base classifier, then, randomly selected samples from the training datasets were used to build the meta-learning task, and the base classifier was further updated using MAML; further, the optimal classifier was achieved by fine-tuning. The rest of this paper is organized as follows: Section 2 introduces the basic concepts of few-shot learning and MAML. In Section 3, a few-shot fault diagnosis model for wind turbines based on MAML is proposed. In Section 4, the on-site wind turbine datasets are input into the proposed model for training and testing, and the results are analyzed. Section 5 concludes the paper.

## 2. Few-Shot Learning and Meta-Learning

### 2.1. Few-Shot Learning Based on Meta-Learning

Meta-learning was originally driven by the human learning process, where humans can learn to recognize a new object with a few instances. The model contains the training set and the testing set. The training set comes from the source domain, the testing set shares the same label space and comes from the target domain, and the source domain does not intersect with the target domain. In the training meta-learning process, *k* data are selected from the training set as the support set *S*, and *q* data as the query set *Q*. If the support set contains *c* categories with k labeled data in each category, the few-shot problem is called a *c*-way *k*-shot. Since the number of labeled samples in the support set is extremely small, meta-learning is performed on the training set to extract transferable knowledge and classify the testing set.

The support set *S* and query set *Q* are extracted from the source domain data, the support set is used as a labeled sample to generate prototype features for the model, and the query set is used as a training sample to update the model. Both the support set and query set form a meta-task, and multiple meta-tasks form a training set. For a *c*-way *k*-shot problem, during the training phase, *c* categories are randomly selected in the training set, and k samples are selected from each category (a total of *k* × *c* data) to construct a meta-task as the support set of the model $S={\left\{\left(x_{i},y_{i}\right)\right\}}_{i=1}^{m}$ (*m* = *k* × *c*); then a batch of samples from the remaining data in these *c* categories are selected as the query set $Q={\left\{\left(x_{i},y_{i}\right)\right\}}_{i=1}^{n}$ (*n* = *q* × *c*) to update the model.

During the training process, different meta-tasks are sampled for each training, so overall, the training contains different combinations of categories, and this mechanism enables the model to learn common parts of different meta-tasks, such as how to extract important features and compare sample similarities. The models learned through this learning mechanism will perform better at classifying when facing new unseen meta-tasks.

### 2.2. Model-Agnostic Meta-Learning

Finn et al. proposed model-agnostic meta-learning (MAML) [40], which is compatible with any model trained with gradient descent, by explicitly training the parameters of the model so that a new task requires only a small number of gradient steps and a small amount of training data to produce good generalization performance. The method has achieved good performance in computer vision [41,42,43], speech recognition [44,45], and reinforcement learning [46].

**Figure 1:**
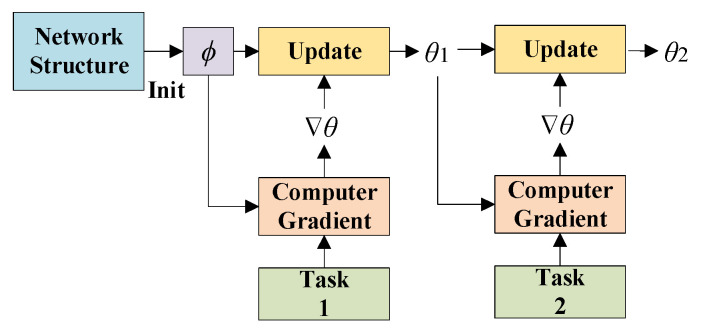
MAML update process.

The MAML meta-gradient update involves a gradient through a gradient, i.e., MAML is based on a secondary gradient, which provides many flexibilities for MAML to adapt to different models. The MAML update process is shown in Figure 1. Define the model as *f*, the parameter of the model as *ϕ*, and its initialization parameter as *ϕ*_0_. For discrete classification tasks with a cross-entropy loss, the loss is:

(1) $$L_{T_{i}}(f_{\phi })=\sum_{x^{\left(j\right)},y^{\left(j\right)}\sim T_{i}}y^{\left(j\right)}\log f_{\phi }(x^{\left(j\right)})+(1-y^{\left(j\right)})\log(1-f_{\phi }(x^{\left(j\right)}))$$

where *x^(j)^*, *y^(j)^* are an input/output pair sampled from task *T_i_*.

**Figure 2:**
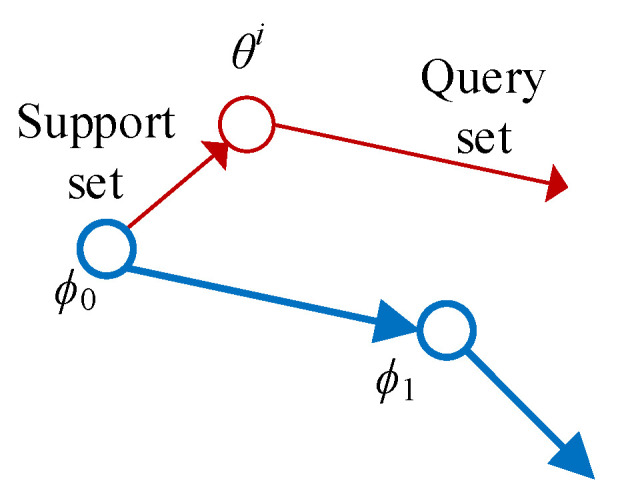
Update gradient by gradient.

Figure 2 illustrates the process of MAML update step by step, assuming that the learning rate for a single task *θ* update is *γ* and the learning rate for model *ϕ* update is *η*, the steps of MAML are as follows:

(1) For task *θ_i_*, compute the gradient on the support set *S* and update the parameters:

(2) $$\theta_{i}{}^{\prime }=\theta -\gamma \nabla_{\theta }L_{T_{i}}(f_{\theta })$$

(2) Calculate the sum of the losses of all tasks on the query set:

(3) $$L(\phi )=\sum_{T_{i}\sim p(T_{i})}L_{T_{i}}(f_{\theta_{i}{}^{\prime }})$$

(3) Update the initialization parameters:

(4) $$\phi \leftarrow \phi -\eta \nabla_{\phi }L(\phi )$$

**Figure 3:**
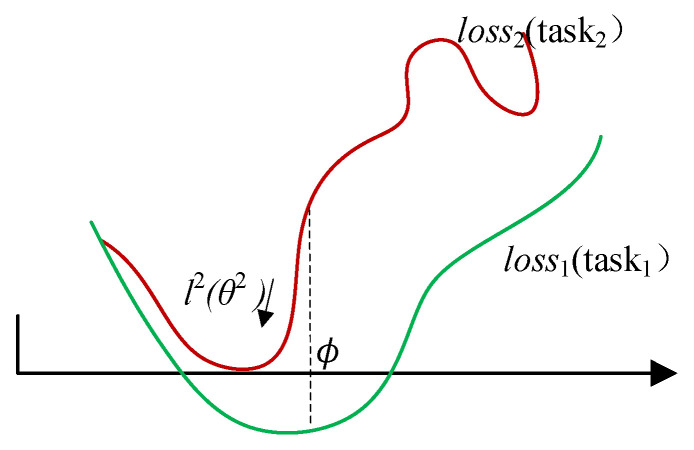
Descend to the global optimum.

As shown in Figure 3, the original intention of MAML is to find the appropriate parameter *ϕ* that makes it possible to descend to the global optimum regardless of the loss curve of task_1_ or task_2_.

### 2.3. Fine-Tuning the Model

Due to the bias in the distribution of the source and target domains, direct classification of the target domain by the base model trained in the source domain usually does not achieve the desired effect. Fine-tuning the pre-trained model using the support set data in the target domain will be beneficial to further improve the classification accuracy of the test set by fine-tuning the parameters of the fully connected layer or the top few layers of the base model. Howard et al. proposed a general fine-tuning language model by varying the learning rate [47]. Nakamura et al. used an adaptive gradient optimizer for fine-tuning while using a lower learning rate during the few-shot retraining [48]. Gao et al. proposed a few-shot fine-tuning method (LM-BFF) for fine-tuning based on language model cues [49]. Chua et al. provided risk bounds on the best predictor found by fine-tuning via gradient descent [50].

## 3. Proposed MAMB for Few-Shot Fault Diagnosis

**Figure 4:**
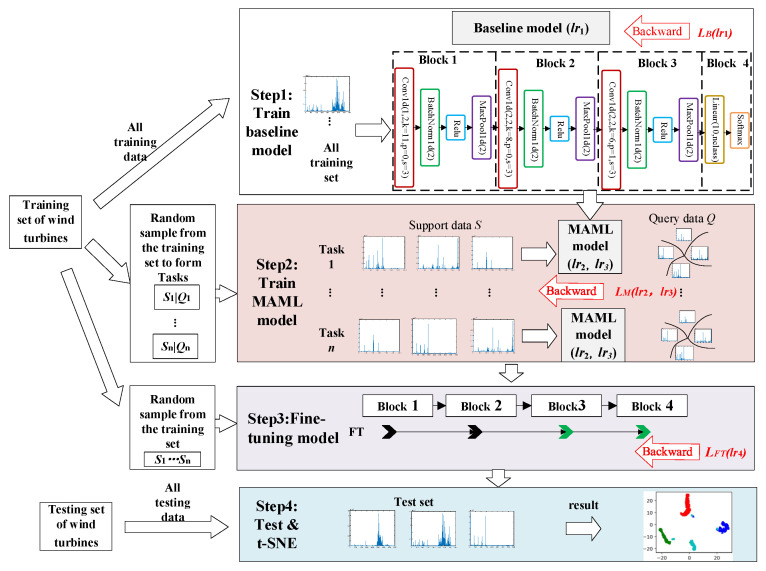
The proposed model (MAMB) for few-shot learning of wind turbines.

In this paper, based on MAML, we proposed a model named model-agnostic meta-baseline (MAMB), which performs few-shot fault detection for multiple faults of wind turbine generator bearings and gearboxes, and the model structure is shown in Figure 4. A small number of existing fault samples of the wind turbine were used to build a meta-learning model, and the model was updated through meta-tasking, which could effectively detect the faults when the same faults occurred again.

The classifier model contained three convolution layers, three BatchNorm1d, three MaxPool1d, and one fully connected layer. The number of neurons in each layer is marked in Figure 4. The activation functions of all layers were rectified linear units (Relu), except for the last layer where the activation function was Softmax. All the data went through the fast Fourier transform, and then it was fed into the model.

The fault diagnosis model was divided into the following steps:

In the first step, the baseline model was trained. All the training set data were input into the classifier model, set the model as *f*, and updated the base model parameters with the learning rate *lr*_1_ as 0.01.

In the second step, the meta-learning model was trained. Assuming a *c*-way *k*-shot learning task, *k* pieces of data of each class were randomly selected from the training data as the support set *S*, another *q* pieces of data were selected as the query set *Q*, and the support set and query set formed a meta-learning task. *N* meta-learning tasks were constructed. The initial parameters of the MAML model were selected from the trained baseline model, and each task was used to update the MAML parameters. The updated learning rate of each task was *lr*_2_ as 0.002, and the updated learning rate of MAML was *lr*_3_ as 0.001.

In the third step, the meta-learning model was fine-tuned. We randomly selected data from the training data to fine-tune with a learning rate *lr*_4_ of 0.0005. As shown in Figure 4, this paper only fine-tuned the last two blocks (green) and froze the first two blocks (black).

In the last step, the test set data were fed into the fine-tuned model for classification and solved for accuracy. The feature embedding was visualized by *t*-distributed stochastic neighbor embedding (*t*-SNE) to test the effectiveness of the proposed model.

Backpropagation updates from the first step to the third step are carried out according to Equation (5).

(5) $$L_{T_{i}}(f_{\phi })=\sum_{x_{i},y_{i}\sim T_{i}}\left[y_{i}\log f_{\phi }(x_{i})+(1-y_{i})\log(1-f_{\phi }(x_{i}))\right]$$

The complete algorithm flow is shown in Algorithm 1.

**Algorithm 1.** few-shot (c-way-k-shot) fault diagnosis based on MAMB.**Input:** Input training data $Tr={\left\{\left(x_{i},y_{i}\right)\right\}}_{i=1}^{M}$, testing data $Te={\left\{x_{i}\right\}}_{i=1}^{I}$, classified model *f*, model parameters *ϕ*, meta-learning model task parameters *θ*, updated learning rate *lr*_2_ of task *θ*, updated learning rate *lr*_3_ of model *ϕ*, updated learning rate *lr*_4_ of fine-tuning. ########################(1) Pre-training baseline models #################### 1: For each training epoch, do: 2:   For each batch, do: 3:     *c_i_* = *f_ϕ_*(*x_i_*) 4:     Backward propagation (with the learning rate as *lr*_1_): $L(f_{\phi })=\sum_{x_{i},y_{i}\sim S_{Si}}\left[y_{i}\log f_{\phi }(x_{i})+(1-y_{i})\log(1-f_{\phi }(x_{i}))\right]$ 5: end ########################(2) train MAML models ############################ 6: Randomly draw data from *Tr* to form *N* tasks, each task containing *k* support sets and *q* query sets, to form $\left\{(S_{1},Q_{1}),(S_{2},Q_{2}),\cdots ,(S_{n},Q_{n})\right\}$ 7: For each training, do: 8:   For each batch, do: 9:     *c_i_* = *f_θ_* (*S_i_*), *l* = *y_i_*log*c_i_* +(1 − *y_i_*)log(1 − *c_i_*) 10:     Update parameters $\theta^{i}=\theta^{i}-lr_{2}\nabla_{\phi }l$ 11:     *c_qi_* = *f_θ_* (*Q_i_*) 12:     *l^n^*(*ϕ*)= *y_i_*log*c_qi_* +(1 − *y_i_*)log(1 − *c_qi_*) 13:     Calculate $L(\phi )=\sum_{n=1}^{N}l^{n}(\phi )$, Backward Propagation: $\phi \leftarrow \phi -lr_{3}\nabla_{\phi }L(\phi )$ 14: end ###################### (3) Fine-tuned meta-learning model ################## 15: Randomly draw data *S_i_* from *Tr* 16: For each training, do: 17:   *c_i_* = *f_ϕ_* (*S_i_*) 18:   Backward Propagation: $\phi \leftarrow \phi -lr_{4}(\nabla_{\phi }L(\phi ))$ 19: end ###################### (4) testing results and t-SNE ######################### 20: For the test set, calculate *c_Ti_* = *f_ϕ_* (*Te_i_*), calculate the accuracy, and draw the *t*-SNE diagram. **Output**: optimized meta-learning model and testing results.

As the working conditions of wind turbines are randomly changing, the working conditions are not stable and constant for the data of wind turbines over a period of time, and the working conditions of the data are unknown. Therefore, in this paper, we took the first 15 data (the first 15 data span a short period of time and could be considered as a constant condition) as training data and the next 240 pieces of data as testing data. While the testing set had unknown conditions (perhaps the same conditions as the training set, or perhaps not), this paper does not make a specific subdivision of the source and target domains. It only solves the results of a large number of testing data when the training model had only a small amount of data in a single working condition.

The update function used in this model was Adam, with 100 training epochs for the pre-trained base model, 200 training epochs for the meta-learning update, and 100 training epochs for the fine-tune. The batch size was 32. In the MAML training step, the sample size of the query set was 5.

## 4. Case Analysis

In this section, three few-shot learning cases are analyzed to verify the advantages of the proposed model, including the few-shot case of the bearing data from Case Western Reserve University (CWRU), the few-shot case of wind turbine generator bearing, and the few-shot case of wind turbine gearbox.

All the three types of data were vibration data. Case 1 was the bearing data from the Case Western Reserve University (CWRU) data, selected from 12DriveEndFault, with operating conditions of 1730, 1750, and 1772 rpm, a sampling frequency of 12 kHz, and a sampling time of 1 s. Case 2 was wind turbine generator drive-end bearing vibration data from field operation, with a sampling frequency of 25,600 Hz and a sampling time of 1 s. Case 3 was wind turbine gearbox vibration data from field operation, with a sampling frequency of 25,600 Hz and a sampling time of 1 s. The input channels provided to the model were the x and y directions of the vibration data.

To further validate the proposed model of MAMB, we compared it with some few-shot or transfer learning algorithms, such as CNN, the Siamese net [7], and the MAML net [40]. To make a fair comparison, we used the same datasets, the same data preprocessing methods (fast Fourier transform), the same classified model, the same epochs, and the same learning rates. Three case studies with one-shot, five-shot, and ten-shot settings were conducted.

### 4.1. Case 1: Fault Diagnosis of CWRU Datasets

In this case, a few-shot fault diagnosis of the CWRU datasets in the drive end was conducted. The available samples are shown in Table 1. The samples contained one category of health data and three kinds of fault data, and each category contained 260 data.

**Figure 5:**
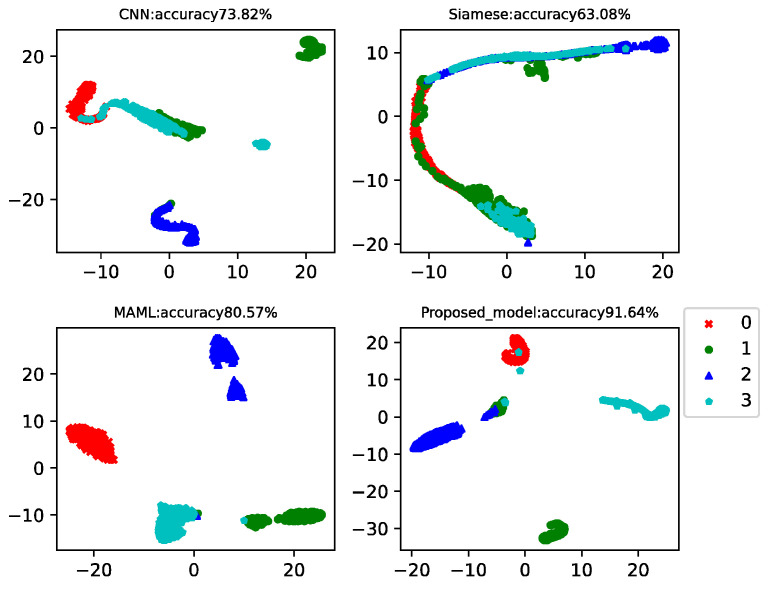
Four-way-one-shot diagnosis of the CWRU data for different algorithms.

**Figure 6:**
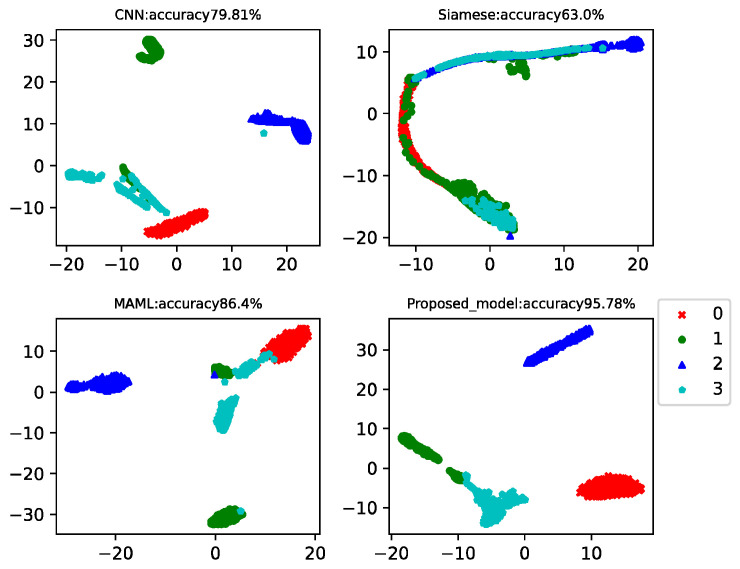
Four-way-five-shot diagnosis of the CWRU data for different algorithms.

**Figure 7:**
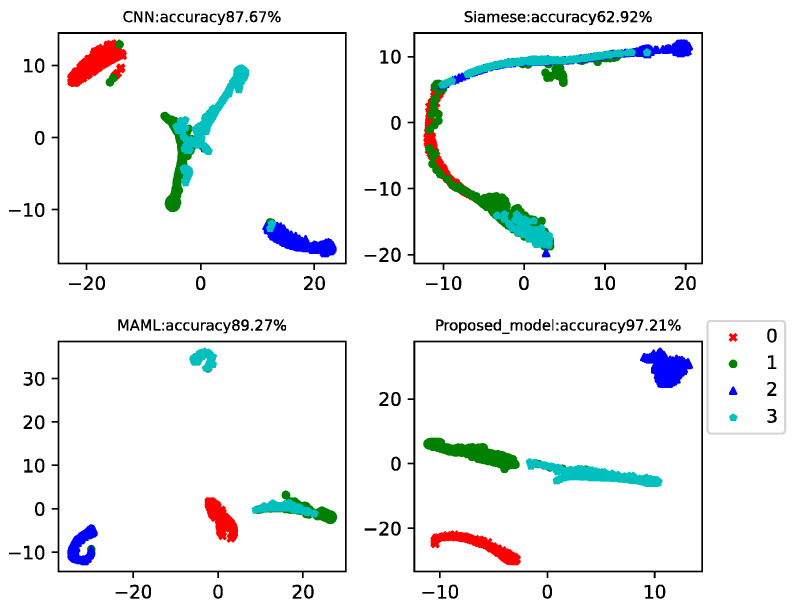
Four-way-ten-shot diagnosis of the CWRU data for different algorithms.

In practical working conditions, healthy data is easy to collect, but fault data is difficult. Therefore, in this case, there were 150 data in the health data training set and 15 data in each of the three faults. Data from the 20th to the 260th of each class was used as a testing set to test the model classification accuracy. This example analyzed the results of four-way-one-shot, four-way-five-shot, and four-way-ten-shot, respectively, and compared with CNN, the Siamese net [7], and the MAML net [40]. The final *t*-SNE is shown in Figure 5, Figure 6 and Figure 7. The accuracy is displayed at the top of each chart.

The fault classification accuracy of the different algorithms using the CWRU dataset is shown in Table 2. The proposed model MAMB already showed relatively high classification accuracy (91.64%) in the four-way-one-shot while reaching 95.78% and 97.21% in the four-way-five-shot and four-way-ten-shot, respectively. The average accuracy was 14.4% higher than that of CNN, 21% higher than that of Siamese net, and 9% higher than that of MAML.

### 4.2. Case 2: Fault Diagnosis of Generator Bearings for Wind Turbines

In this case, a few-shot fault diagnosis of the generator bearings for wind turbines was conducted. The available samples are shown in Table 3.

The generator bearing data for the wind turbine included health data and three types of faults, and each category contained 260 data. The latter two faults were compound faults. In the actual operating conditions, wind turbines mostly have compound faults, and this paper studies the few-shot problem of compound faults, which has better engineering significance. At the same time, the operating conditions of wind turbines are changing at any time, and the first 15 data were taken for training in this paper. Usually, the latter 240 data are in different operating conditions from the training data. The model could also be further tested for different operating conditions.

**Figure 8:**
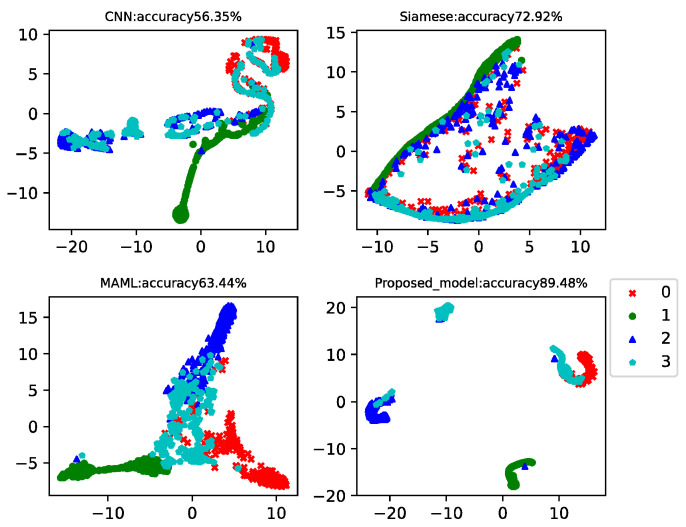
Four-way-one-shot diagnosis of the generator bearing data for the wind turbines using different algorithms.

**Figure 9:**
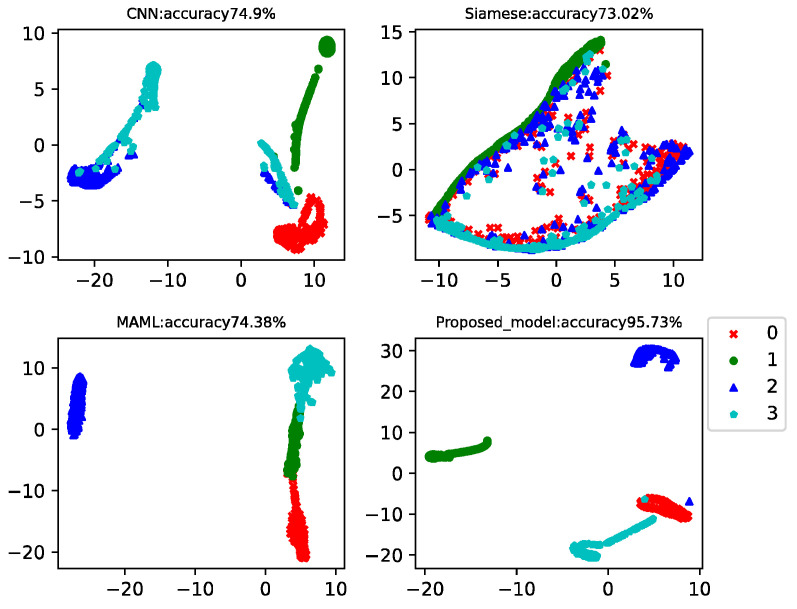
Four-way-five-shot diagnosis of the generator bearing data for the wind turbines using different algorithms.

**Figure 10:**
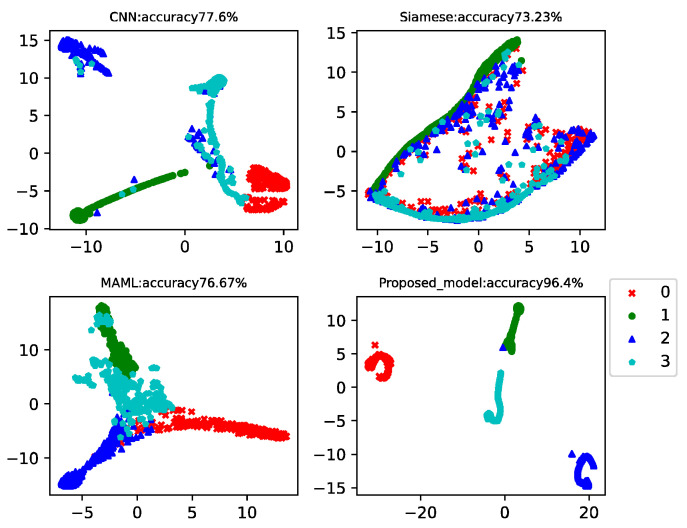
Four-way-ten-shot diagnosis of the generator bearing data for the wind turbines using different algorithms.

This case analyzed the results of four-way-one-shot, four-way-five-shot, and four-way-ten-shot, respectively, and compared with CNN, the Siamese net [7], and the MAML net [40]. The final *t*-SNE is shown in Figure 8, Figure 9 and Figure 10. The accuracy is displayed at the top of each chart.

The fault classification accuracy of the generator bearings for wind turbines using different algorithms is shown in Table 4. The proposed MAMB model showed relatively high classification accuracy (89.48%) in the four-way-one-shot while reaching 95.73% and 96.4% in the four-way-five-shot and four-way-ten-shot, respectively. The average accuracy was 24% higher than that of CNN, 21% higher than that of Siamese net, and 22% higher than that of MAML.

As the operating conditions of wind turbines change all the time, it can be seen that the classification accuracy of CNN, Siamese net, and MAML was much lower than that of the CWRU data. However, the proposed model incorporated the basic classification advantages of CNN and the learning advantages of MAML, and the test accuracy still reached consistently high values.

### 4.3. Case 3: Fault Diagnosis of Wind Turbine Gearbox

This case focused on a few-shot fault diagnosis of the gearbox of wind turbines. The available gearbox samples are shown in Table 5, and the samples contained one category of health data and four kinds of fault data, each category contained 260 data. Fault 2 is a compound fault.

**Figure 11:**
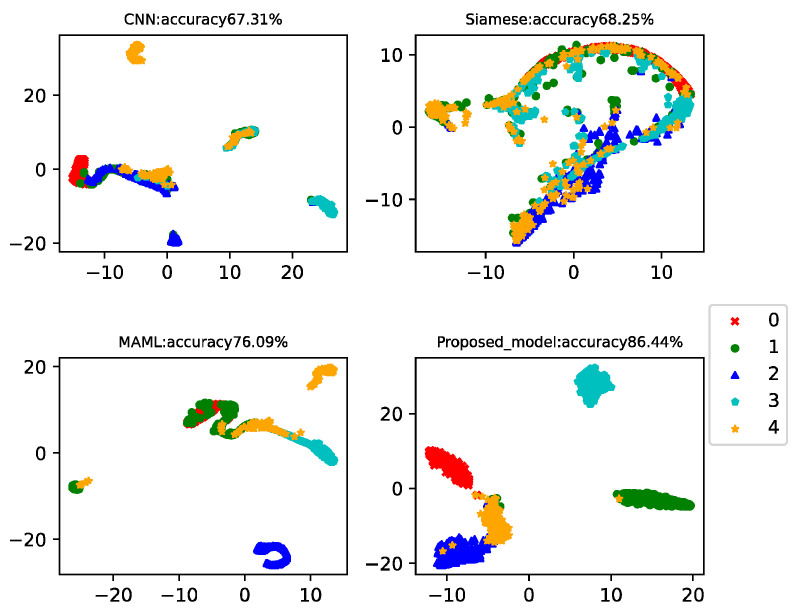
Five-way-one-shot diagnosis of the wind turbine gearboxes using different algorithms.

**Figure 12:**
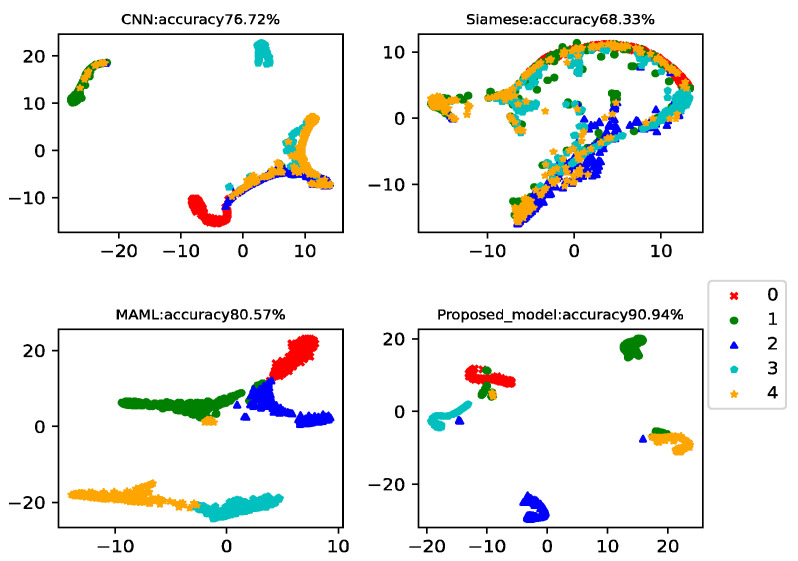
Five-way-five-shot diagnosis of the wind turbine gearboxes using different algorithms.

**Figure 13:**
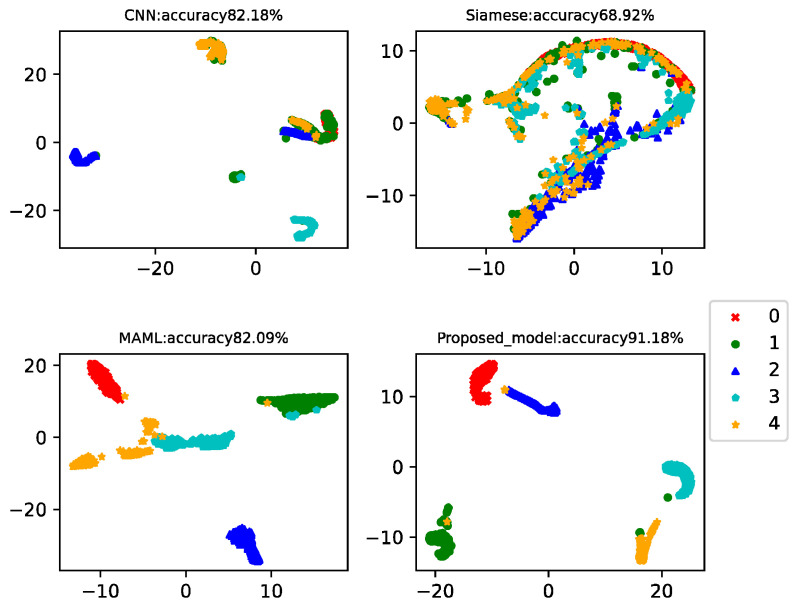
Five-way-ten-shot diagnosis of the wind turbine gearboxes using different algorithms.

In this example, there were 150 health data in the training set and 15 data for each type of failure. Data from the 20th to the 260th of each class was used as a testing set to test the model classification accuracy. This example analyzed the results of five-way-one-shot, five-way-five-shot, and five-way-ten-shot, respectively, and compared with CNN, the Siamese net [7], and the MAML net [40]. The final t-SNE is shown in Figure 11, Figure 12 and Figure 13. The accuracy is displayed at the top of each chart.

The fault classification accuracy of wind turbine gearboxes using different algorithms is shown in Table 6. The proposed model reached 86.44%, 90.94%, and 91.18% in the five-way-one-shot, five-way-five-shot, and five-way-ten-shot, respectively. The average accuracy was 14% higher than that of CNN, 21% higher than that of Siamese net, and 10% higher than that of MAML.

### 4.4. The Impact of the Number of Training Data on the Results

This section analyses the effect of the number of training samples on the results using MAMB, the accuracy result was shown in Table 7. The sample sizes of each class were 15 or 20. It can be seen that the accuracy of the model improved as the number of training samples increased. 

## 5. Conclusions

Fault diagnosis of wind turbines plays an important role in improving the reliability of wind turbines. However, the operating conditions of wind turbines change randomly, and multiple faults often occur simultaneously. When fault samples are small, ordinary deep learning can fall into overfitting, which in turn leads to low diagnostic accuracy.

Model-agnostic meta-baseline (MAMB)-based few-shot learning was presented in this paper to achieve the few-shot diagnosis of compound faults of the wind turbines drivetrain under variable operating conditions. The model consists of four steps: pre-training the base model, training the MAML, fine-tuning, and testing.

This paper analyses the diagnosis of one-shot, five-shot, and ten-shot tasks of single and compound faults in CWRU, wind turbine generator bearings, and wind turbine gearboxes. It was also compared with other algorithms to verify the accuracy and stability of the proposed method. The results are also presented by *t*-SNE.

The proposed model MAMB combines the advantages of CNN in basic classification and MAML in learning new tasks. The results show that the proposed model MAMB was superior to CNN, Siamese net, and MAML in the classification accuracy of three kinds of data. Especially for wind turbine data, the accuracy of the proposed model MAMB was higher than that of other models. This shows that the proposed model could solve the problems of wind turbine variable operating conditions and composite diagnosis better.

In the future, the recognition of unknown classes of wind turbines should be further considered through transfer learning.

## Figures and Tables

**Table 1:** Fault description of CWRU data.

Fault Type	Label	Number of Samples from the Training Set	Number of Samples from the Testing Set
Healthy	0	100	240
Outer Race 6	1	15	240
Inner Race	2	15	240
Rolling fault	3	15	240

**Table 2:** Comparison of MAMB with different algorithms in the few-shot diagnosis of the CWRU data.

Algorithms	4-Way-1-Shot	4-Way-5-Shot	4-Way-10-Shot	Average
CNN	73.82%	79.81%	87.67%	80.43%
Siamese net [7]	63.08%	63.0%	62.92%	63%
MAML [40]	80.57%	86.4%	89.27%	85.41%
MAMB (proposed model)	**91.64%**	**95.78%**	**97.21%**	**94.88%**

**Table 3:** Fault description of generator bearings for wind turbines.

	Fault Type	Label	Number of Samples from the Training Set	Number of Samples from the Testing Set
Healthy	No faults	0	100	240
Fault 1	Outer ring failure	1	15	240
Fault 2	Inner ring failure + Outer ring failure	2	15	240
Fault 3	Inner ring failure + Rolling failure + Cage failure	3	15	240

**Table 4:** Comparison of MAMB with different algorithms in the few-shot diagnosis of the generator bearings for wind turbines.

Algorithms	4-Way-1-Shot	4-Way-5-Shot	4-Way-10-Shot	Average
CNN	56.35%	74.9%	77.6%	69.62%
Siamese net [7]	72.92%	73.02%	73.23%	73.06%
MAML [40]	63.44%	74.38%	76.67%	71.5%
MAMB (proposed model)	**89.48%**	**95.73%**	**96.4%**	**93.87%**

**Table 5:** Fault description of wind turbine gearbox.

	Fault Type	Label	Number of Samples from the Training Set	Number of Samples from the Testing Set
Healthy	No faults	0	100	240
Fault 1	Spalling of gears in the intermediate shaft	1	15	240
Fault 2	Broken teeth of gears in the intermediate and high-speed shaft	2	15	240
Fault 3	Broken teeth of gears in the high-speed shaft	3	15	240
Fault 4	Broken teeth of gears in the intermediate shaft	4	15	240

**Table 6:** Comparison of MAMB with different algorithms in the few-shot diagnosis of the gearboxes for wind turbines.

Algorithms	5-Way-1-Shot	5-Way-5-Shot	5-Way-10-Shot	Average
CNN	67.31%	76.72%	82.18%	75.4%
Siamese net [7]	68.25%	68.33%	68.92%	68.5%
MAML [40]	76.09%	80.57%	82.09%	79.58%
MAMB (proposed model)	**86.44%**	**90.94%**	**91.18%**	**89.52%**

**Table 7:** The effect of the number of training samples on the results for wind turbines data.

No. of Shot	1-Shot		5-Shot		10-Shot	
No. of Train Data	15	20	15	20	15	20
Bearing (4-way)	89.48%	90.02%	95.73%	96.08%	96.4%	97.2%
Gearbox (5-way)	86.44%	88.25%	90.94%	92.0%	91.18%	92.09%

## Data Availability

https://github.com/yyxyz/CaseWesternReserveUniversityData.
